# Current concepts in developmental dysplasia of the hip and Total hip arthroplasty

**DOI:** 10.1186/s42836-019-0004-6

**Published:** 2019-08-01

**Authors:** Yan Wang

**Affiliations:** Department of Orthopedics, Chinese PLA General Hospital, 28 Fuxing Road, Beijing, 100853 China

**Keywords:** Hip dysplasia, Abnormalities, Reconstruction

## Abstract

Developmental dysplasia of the hip (DDH) is a spectrum of pathology that involves dysplasia of both the acetabulum and the femur. If left untreated, it can develop to hip pain and osteoarthritis, which eventually require total hip arthroplasty (THA). A broad array of anatomical abnormalities of the acetabulum and femur, plus the younger age of DDH patients make THA a great challenge. Meticulous operation planning with various options is one of the most important prerequisites of a successful THA. This review presents the current concepts of acetabular and femoral reconstruction in THA for DDH, including high hip center, acetabular bone deficiency, highly porous metal, correction of femoral anteversion, femoral shortening osteotomy, stem selection, among others.

## Introductions

DDH represents a condition where the “ball and socket” joint of the hip does not properly form in infants and young children. DDH occurs one in every 1000 live births and is more common in girls. It is the most common cause of secondary osteoarthritis in adults under 40 years of age. If left untreated, it can lead to hip pain and osteoarthritis, which eventually require total hip arthroplasty [1]. Because of morphological diversity of deformities, technical difficulties, inadequately designed prostheses, and so on, THA in dysplastic hips, especially in high dislocation, remains a challenging task [2, 3]. In addition, some complications, such as leg length discrepancy (LLD), nonunion at the osteotomy site, nerve injuries, postoperative dislocation of the hip joint, valgus deformities of the knee, and aseptic loosening are still the major DDH-related issues. Therefore, THA for DDH is still a technically demanding surgery that requires an in-depth understanding of the anatomical abnormalities and a good mastery of complex techniques. Some problems concerning both the pelvis and femur also need to be addressed. In this paper, we present the current concepts of acetabular and femoral reconstruction.

### Anatomical abnormalities and classifications

DDH refers to a spectrum of pathology that involves dysplasia of the acetabulum or proximal femur, hip instability and subluxation, and dislocation of the hip joint. Several classification systems have been developed to characterize DDH in adults. The most frequently used system is Crowe classification system [4]. The system is a quantitative method based on the amount of femoral head subluxation in relation to the height of the undeformed femoral head, not informative on the pathoanatomy of the acetabulum. Dysplasia is of four different types (Table 1).

**Table 1:** Crowe classification [1]

Type	Proximal displacement	Femoral head subluxation
Crowe I	< 10%	< 50%
Crowe II	10–15%	50–75%
Crowe III	15–20%	75–100%
Crowe IV	> 20%	> 100%

Another commonly used classification system for DDH in adults was introduced by Hartofilakidis et al. [5]. Different from the aforementioned system, the Hartofilakidis classification relies on the anatomy of the acetabulum based on radiographic appearance of the hip. It lists three types: dysplasia, low dislocation, and high dislocation. (Table 2) Nonetheless, “borderline hips” can be encountered due to an unclear boundary between true and false acetabulum.

**Table 2:** Hartofilakidis classification [5]

Congenital hip diseases	Description	Subtypes
Dysplasia	The femoral head is contained within the original acetabulum despite the degree of subluxation or proximal femoral migration	A
Low dislocation	The femoral head articulates with a false acetabulum that partially covers the true acetabulum to varying degrees	B1 The false acetabulum covers more than 50% of the true acetabulum; resembling dysplasia
		B2 The false acetabulum covers less than 50% of the true acetabulum; resembling high dislocation
High dislocation	The femoral head is completely out of the true acetabulum and migrated superiorly and posteriorly to varying degrees	C1 The femoral head articulates with a false acetabulum
		C2 No false acetabulum; the femoral head is free-floating within the gluteal musculature

An acetabulum of Crowe type I/II or Hartofilakidis type A, with a decreased center-edge angle, is generally considered to be close to a normal one. In cases of high dislocation, true acetabulum tends to be a shallow oval-shaped fossa. And it is commonly perceived that the DDH is associated with lateral and anterior acetabular deficiency, with the acetabulum oriented in abnormal anteversion.

The common deformities in the dysplastic femur include excessive neck version, a posteriorly displaced greater trochanter, a valgus neck-shaft angle, hypoplasia of the intramedullary canal, rotational metaphyseal-diaphyseal mismatch, and contracture of abductor muscles.

Therefore, those malformations entail an individualised strategy of care for each step of the treatment process. Detailed operation planning with various options is one of the most important prerequisites of a successful THA.

### Surgical considerations

A good preoperative planning should include five steps, i.e., evaluating the preoperative LLD, relocating the hip center, predicting the use of shortening osteotomy, choosing appropriate implants, and achieving primary stability.

In our hospital, full-length anteroposterior radiographs of the entire lower limb and spine involved are routinely obtained preoperatively, which helps identify the causes of LLD, such as pelvic obliquity, scoliosis, and a discrepancy either on the length of the femur or tibia, or both. CT scan from hip to knee may help in the assessment of DDH, because the diameter of femoral canal may be overestimated on anteroposterior radiographs and underestimated on lateral radiographs due to rotational mismatch of the metaphysis and diaphysis [6]. In addition, the version and orientation of femur and acetabulum can be observed, which is helpful for individual prosthesis selection.

It should be noted that the small diameter of the bony acetabulum in high dislocated hips typically dictates the use of smaller acetabular component. In spite of the narrow space for cup placement, many authors found that the bone stock at posterior part of the true acetabulum was abundant [7–10]. Besides, due to the great variations of femoral version, mismatch between the proximal and distal parts of the femur can be encountered in the dislocated hips, which may limit the use of conventional double-wedged stems.

There are some other considerations, including the adequate cup coverage, reconstruction of the abductor mechanism and safe reduction of the joint. So it is imperative to resolve the problems with both the acetabulum and femur.

### Key issues in the management of acetabulum in THA

The first issue is acetabular cup position. The location of the placement of the acetabular component defines the new center of hip rotation, which in turn influences hip biomechanics, leg length, and femoral reconstruction. It is biomechanically desirable to place the acetabular cup at the site of the true acetabulum, which can restore the center of hip rotation and attain optimal abductor muscle function. A high but not lateral position may be also acceptable. A high hip center utilizes live host bone, thus avoiding the requirement for bone graft, and it is technically easier than reconstruction with the true acetabulum. However, a number of disadvantages, including incompetence of abductor mechanism, excessive joint reaction force and high dislocation rate, were reportedly associated with high hip center [11, 12]. Several excellent outcomes, as shown by a minimum 10-year follow-up, were accomplished with the hip center placed 24.5–26.8 mm above the inter-teardrop [13–15]. Moreover, in a study of 53 cementless cups inserted in dysplastic hips, with a minimum follow-up of 10 years, the polyethylene wear rate was significantly greater when the cup was positioned lateral to the acetabular teardrop by > 25 mm and aseptic loosening of the femoral component was significantly greater when the cup was placed > 25 mm superior to the teardrop [16]. Apart from the wear, some surgeons also have concern about the gait. Relative findings by Fukui et al. showed only 10% positive Trendelenburg sign, indicating that a high center of hip rotation of up to approximately 30 mm from the inter-teardrop line is a feasible option for patients with DDH. And they also emphasized the use of stems that allow the restoration of femoral offset and the abductor lever arm [17]. So proximal placement of acetabular components might not negatively impact on the outcome of acetabular reconstruction provided that the component was not lateralized.

The second issue is bone stock deficiency, especially at the superolateral part, which is frequently encountered, leaving a portion of the cup uncovered. In order to achieve stability and adequate ingrowth on bone, the cup dictates at least 70% coverage by the native bone [18]. Managing deficient acetabular bone in THA requires intensive thinking and planning. In coping with significant bone deficiency, the alternatives available include acetabular augmentation with cement or bone autograft or porous metal augments, or medialization of the component with or without medial wall osteotomy, or reinforcement ring [19–25].

Bone grafting used to be a popular choice. Bone grafts used for acetabular reconstruction includes morselized bone graft, structural bone graft and hybrid bone graft. Morselized bone grafts are widely used because of the advantages of simple production and short healing time. Structural onlay allografts may provide mechanical stability for the cementless prosthesis and increase bone stock. Some retrospective studies reported higher graft failure rates in cemented acetabular augmentation after 15 years. Aseptic loosening was the number-one cause of revision [26, 27]. A finite element study also demonstrated that rigidly fixed load-transmitting bone graft filling any superolateral bone defect could reduce the stresses at the bone-cement interface of cemented acetabular component [28]. Cemented acetabular components in combination with autogenous bone graft augmentation provides favorable early-to-midterm outcomes, but graft collapse will happen over a long period of time, particularly, when the acetabulum has been filled with a large amount of autogenous cancelous bone grafts and the cup is cemented in position without pressure [18, 29, 30]. Conversely, the use of uncemented cup yielded more favorable results, but initially the long-term results were discouraging [31, 32]. And then, Kim et al. reported a 94% long-term survival of bulk femoral head autograft for acetabular reconstruction in cementless THA for DDH [19]. The result led them to speculate that the high rate of graft incorporation and survival could be attributed to three factors: initial stability of the graft and cup, impacted cancellous surfaces of the host and graft and a porous surface of the largest possible cups.

The medial protrusio technique, developed to maximize bony coverage of the cup, has been reported to provide reasonable midterm results [21–23]. But long-term follow-up of this technique is warranted to prove its durability. What’s more, the acetabular medial wall osteotomy is not recommended when acetabular medial wall thickness is less than 10 mm. If the reinforecement ring is employed, there is no need for cup medicalization and bone graft. Gill et al. reported 33 acetabular reconstructions with reinforcement ring, which were followed up for 6.7 years on average. Only 2 joints were revised due to loosening [33]. However, it might be a project too big for a small acetabulum and increases the complexity of the revision.

The third issue is the use of highly porous metal and it might change the concept of acetabular reconstruction. Trabecular Metal (Zimmer), represented by the next-generation highly porous metals, is characterized by high porosity, ideal pore size, low elastic modulus and high surface roughness, which can provide better bone integration and biological fixation capability, increase the speed and depth of bone growth, and improve initial stability [34, 35]. So it can be speculated that an acetabular cup equipped with highly porous metal is likely to be more competent for the reconstruction of mild to moderate acetabular bone defects, with a portion of the cup uncovered by native bone. Several studies using highly porous metal cups reported satisfactory mid-term clinical and radiographic results, with no cup revised for aseptic loosening [36, 37]. However, as for these expensive implants, long-term follow-up and some health-economic analysis regarding the cost-effectiveness are needed.

### Key issues in reconstruction of proximal femur

The first issue is anteversion angle of femur, which refers to the rotation of the neck of the femur around the diaphysis. A variety of femoral anteversions may happen during surgery. Severe abnormal anteversion can be corrected with derotational subtrochanteric osteotomy, cemented stem, special cementless stems (either modular or conical stems with flutes), and even a customized prosthesis [38–41]. Nevertheless, in the setting of rotational mismatch of the metaphysis and diaphysis, conventional tapered stems may be under-sized, which may cause malalignment or malrotation of implantation, and even femoral fracture [6].

The second issue is shortening of the femur. During THA for DDH with a high dislocation (Crowe type IV), femoral osteotomy and shortening make reduction easier. So far, proximal, subtrochanteric and distal osteotomies have been described for femoral shortening.

Characteristically, subtrochanteric osteotomy can rotate the proximal fragment so that the metaphyseal flare and the greater trochanter are placed into a more anatomic position, which helps restore the function of abductors. Several techniques are commonly used, including transverse, oblique, z-shaped, and the double chevron osteotomy [42–45]. Among them, transverse subtrochanteric osteotomy is less reliable than other more complicated techniques. In a biomechanical study, however, Muratli et al. [46] found no difference with regard to stability of the four techniques. A variety of methods are developed to reinforce the osteotomy site, including the use of K-wire, cable, cerclage bands, and plate with or without the resected bone segment [47, 48].

Proximal osteotomy includes greater trochanteric osteotomy or a trochanteric slide osteotomy, and trochanteric osteotomy that refers to sequential resection of femoral neck. Greater trochanteric osteotomy is indicated when greater trochanteric repositioning is desirable to restore hip biomechanics. The possible complication is nonunion at the osteotomy site [49]. Trochanteric osteotomy allows small, incremental resection of the femoral neck. And a large amount of resection of the proximal femur compromises the structure of femoral calcar, which usually leaves the remaining femur a straight tube with little metaphysis flare to mate with the stem. Under this circumstance, a small, cemented stem or a cone-shaped stem can be inserted. Besides, this technique makes the great trochanter lie well above the hip center, without increasing the power of abductor mechanism. So, in our opinion, a small amount of resection is acceptable, and it can serve as a supplement to great trochanteric or subtrochanteric osteotomy. Though rare, distal osteotomy, which is also called middiaphyseal osteotomy, is indicated when there is a concomitant severe valgus deformity of the knee [50].

So far, researchers fail to agree on the appropriate amount of resection [51–54]. Conversely, some methods were reported to be able to achieve hip reduction without subtrochanteric osteotomy, such as iliofemoral distraction before THA, intraopetaive injection of muscle relaxant and leverage [55–57]. However, trochanteric osteotomies were performed in some of their cases, and some patients classified as Crowe type III with less severe deformities were also included in their series. As we see it, most of the current researches available are short of homogeneity. And due to all sorts of the morphologic abnormalities and soft tissue condition, there might not exist an all-mighty technique which can deal with all the complicated cases. Surgeons should take multiple factors into consideration, including the leg length discrepancy before surgery, the flexibility of the spinopelvic complex, the function of abductor mechanism, and, the last but not the least, patient’s proprioception about the LLD [58–62].

The third issue is the selection of a proper stem. Cemented stem can adapt to the abnormal anteversion and intramedullary canal to a certain degree. However, the cement might leak into the osteotomy site when used in subtrochanteric osteotomy. In the late 1980s, cementless stems were introduced and steadily gained its popularity all over the world. Cementless fixation relies on primary press-fit stability and subsequent bone ongrowth or ingrowth, which is particularly suitable for young patients with high activity demand. Besides, although excellent outcomes were attained in cases treated with fully-coated stems, the problem of proximal stress shielding remains [63]. And Perka et al. reported a rate of 5.8% of intraoperative fractures of the proximal femur [64]. The classical stem designs, which are canonized by most surgeons, are modular stems and conical stems with flutes. Both of them can be rotated to neutralize the native abnormal anteversion and mitigate the need for a derotational femoral osteotomy.

Modular stems maximally allow for both anatomical and biomechanical reconstruction. Modular stems can decouple the metaphyseal/diaphyseal fit from the different versions of prosthetic components, even, in excessive neck-shaft angle or straight intramedullary canal. It allows a surgeon to implant the sleeve in opposite direction or to use a cone-shaped sleeve, and to adjust length and offset of the femur. Moreover, the beauty is that the modular stem represented by S-ROM (Depuy) can provide excellent angular and rotational stability [65, 66]. The downsides are the high price, weaker strength of the modular junctions and fretting or corrosion of interface [67–69].

Conical stems with flutes produced by Wagner Cone (Zimmer) can fit into the cylindrical intramedullary canal and the anteversion can also be adjusted. The conical design of the stem and the eight longitudinal ribs provide good rotational stability [70]. One of the complications is subsidence of stem, which is governed by a number of factors, especially in physically-active and obese patients [70–72].

Deformity of the proximal femur and the narrow diaphyseal canal occasionally precludes the use of an off-the-shelf stem. Customized stems are designed and manufactured principally for patients with abnormal size and shape of the proximal femur. Drawbacks include the protracted production time and the high manufacturing cost. Worst of all, it may well put the surgeon in a dilemma if the limited options available fail to work during operation.

## Conclusions

A broad range of pathomorphologic changes of the acetabulum and femur, and the diverse and often younger age spectrum of DDH patients make the THA a great challenge for surgeons. Currently, THA generally yields excellent results in DDH population, but some complications, such as leg length discrepancy, abnormal gait, and knee valgus, remain major concerns. In the future, in-depth studies of anatomical morphology should be conducted to ensure optimal preoperative planning. Future development in artificial intelligence and portable facilities may help measure and rebalance neuromuscular tension better. More biocompatible and durable materials will be used for reconstruction of the hip joint.

## Data Availability

Data sharing not applicable to this article as no datasets were generated or analyzed during the current study.

## Abbreviations

DDH: Developmental dysplasia of the hip
LLD: Leg length discrepancy
THA: Total hip arthroplasty
